# Understanding health literacy and digital healthy diet literacy in rural women in Türkiye: a cross-sectional study on social media use and Mediterranean diet adherence

**DOI:** 10.3389/fpubh.2025.1559159

**Published:** 2025-05-30

**Authors:** Zehra Batu, Sefer Kalaman, Mikail Batu, Zülfiye Acar Şentürk, Handan Güler İplikçi, Nazmi Ekin Vural, İlkay Burak Taşkıran

**Affiliations:** ^1^Department of Nutrition and Dietetics, Nezahat Kelesoglu Faculty of Health Sciences, Necmettin Erbakan University, Konya, Türkiye; ^2^Faculty of Communication, Department of New Media and Communication, Ankara Yıldırım Beyazıt University, Ankara, Türkiye; ^3^Faculty of Social and Human Sciences, Department of Public Relations and Advertising, Necmettin Erbakan University, Konya, Türkiye; ^4^Faculty of Communication, Department of Public Relations and Advertising, Yozgat Bozok University, Yozgat, Türkiye; ^5^Faculty of Communication, Department of Public Relations and Promotion, Manisa Celal Bayar University, Manisa, Türkiye; ^6^Faculty of Communication, Department of New Media and Communication, Yozgat Bozok University, Yozgat, Türkiye; ^7^Faculty of Communication, Department of Public Relations and Advertising, İstanbul Yeni Yüzyıl University, Istanbul, Türkiye

**Keywords:** health literacy, digital healthy diet literacy, social media, women, Mediterranean diet

## Abstract

**Background:**

Health literacy (HL) is the ability to search for, acquire, understand, interpret, and act upon basic information, concepts, and services about health to make correct and informed health decisions about. In terms of public health, low HL can lead to negative health outcomes, increased healthcare costs, increased medical and medication errors, disruption of the treatment process, and increased mortality.

**Methods:**

This study investigated factors associated with HL and digital healthy diet literacy (DDL), focusing on rural women using social media. This cross-sectional study included women aged 18–65 years living in rural areas in Türkiye between 01/12/2023 and 29/02/2024. The variables examined regarding HL and DDL are duration and purpose of use of the internet and social media, compliance with the Mediterranean diet, and sociodemographic characteristics. HL and DDL were evaluated using the Turkish version of HLS-SF12 and DDL scale.

**Results:**

The maximum score that can be obtained from the HLS-SF12 and DDL scale is 50 and the average index values of the participants were determined as 26.70 and 21.99, respectively. HL and DDL index scores were affected by the purpose and duration of internet/social media use. HL and DDL index scores were found to be higher in those who had a diet history under dietitian counseling, university graduates, those who adhered to the Mediterranean diet, and those with higher household income. In addition, it was determined that DDL index and HL index correlated with Mediterranean Diet Adherence Screener (MEDAS) score and Body Mass Index (BMI).

**Conclusion:**

HL index and DDL index scores differ depending on the duration and purpose of internet use, age, presence of chronic disease, education level, and income level. It is recommended to conduct further studies evaluating the use of social media as a tool to promote HL, DDL, and healthy eating behaviors in different populations.

## Introduction

Health literacy (HL), which is part of the promotion and development of healthy living, refers to an individual’s ability to access, understand, evaluate, and effectively use comprehensible and reliable health information and services (1, 2).

The level of HL, which is important for ensuring the health development of individuals and society, may vary according to social, economic, technological, and demographic characteristics (3). The place where a person lives is undoubtedly one of the most determining factors of these characteristics. This is because the geographical region in which an individual lives and the cultural structure in which they grow up directly affects their level of health literacy (4). So much so that in most studies, it has been determined that the health literacy level of the rural population is lower than the urban population. Likewise, from a gender perspective, it has been observed that rural women have lower health literacy levels than urban women (5).

Women’s health literacy is very important in terms of affecting both their own health and the health of their family and society (6). When women’s health literacy is considered from the perspective of Türkiye, it is possible to say that the desired level has not yet been reached (4). Limited, problematic, or insufficient health literacy has both individual effects, such as an unhealthy lifestyle, insufficient knowledge about chronic diseases, lack of understanding of medical training, and incorrect use of medication, and effects on society, such as increased emergency room use and hospitalizations, higher health expenditure, and higher death rates (7). In Türkiye, 72.4% of women have inadequate, problematic, or limited health literacy (8).

Social media is a powerful tool when used to deliver accessible, understandable, and effective health information to large audiences. In addition, social media is considered an effective and alternative way to increase the health literacy levels of low-income and rural people (9). Empirical studies have also revealed that people frequently use social media to collect and share online health information and increase their health literacy levels (10). Moreover, there are also studies showing that individuals with high social media use skills also have high health literacy levels (11). Therefore, it is important to ensure that women, especially those living in rural areas, use social media more effectively and popularize the use of digital technology in order to increase their health literacy levels (12).

As digital access increases in rural areas, opportunities to obtain digital health information also increase (13). For example, rural women learn about diet and healthy lifestyle habits through social media, an increasingly popular medium for obtaining health information (14). The process of obtaining information is essential for developing digital health literacy (14). Rural women have limited access to health services and often play a central role in the family’s health care. Therefore, studies on women’s health literacy and digital healthy diet literacy have the potential to affect not only women’s health but also the health, healthy food preferences, and health practices of the entire family (15, 16).

This study aimed to determine the relationship between social media usage levels and health literacy levels, digital healthy diet literacy (DDL) levels, and healthy eating behaviors among rural women using social media in Türkiye. Therefore, this study focuses on the following questions: What are the levels of HL and DDL in rural women? Do the duration and purpose of Internet and social media use affect the levels of HL and DDL? Are HL and DDL levels associated with healthy eating habits?

## Materials and methods

### Sampling principles

The research population consisted of 4.498.559 rural women between the ages of 18–65 in rural areas in Türkiye. The sample size was calculated with $n=Np^{2}Z^{2}÷[(N-1\;x\;t2)+(p\;x\;q\;x\;Z^{2})]$ formula (*N* = Population, *n* = Number of samples, *p* = Frequency of occurrence of the feature, q = Frequency of not seeing the feature we are interested in, Z = Standard value according to confidence level, t = Tolerable error) (17).

Accordingly, the sample size was calculated as 384 people with a 95% confidence level and a 5% margin of error. Considering the possibility of data loss or volunteers withdrawing from the study, twice the minimum number, the target was to reach 768 people who met the inclusion criteria.

The sample was determined based on the Statistical Regional Units Classification (NUTS1) created by the Turkish Statistical Institute and the State Planning Organization, and the purposeful sampling method was used. Within the scope of NUTS1, the provinces with the lowest population density and share of gross national product in each region and with a rural population were selected. Istanbul was excluded from the list because it is a metropolitan city, does not have a rural area, and has a high share of the gross national product. For the study sample, 11 provinces (Ağrı, Afyon, Batman, Bitlis, Düzce, Edirne, Giresun, Karaman, Osmaniye, Tokat, and Yozgat) representing the rural population from different geographical regions of Türkiye were selected. In this way, the effect of different geographical conditions experienced by rural women on the findings of the study was taken into account.

In addition, in order to provide diversity in the study, settlement areas of different sizes (villages, towns, sub-districts, etc.) were selected in the provinces in order to reflect the socioeconomic differences in the rural areas where the target audience lives.

***Inclusion criteria***: Being a volunteer, being a woman, being between the ages of 18–65, residing in a residential area classified as rural for at least 2 years, being literate, having at least one social media account, and using social networks at least once a week.

***Exclusion criteria***: Having visual or hearing problems, having a healthcare profession (physician, nurse, pharmacist, dietitian, etc.), or filling out surveys incompletely.

### Data collection

A survey technique was used in the research. The announcement of the study was made social media accounts, mukhtars’ offices, and flyers. Individuals who volunteered to participate in the study and reached out to the researchers were evaluated in terms of compliance with the inclusion and exclusion criteria. Appointments were made with suitable volunteers, and the surveys were conducted face to face between 01/12/2023 and 29/02/2024 by trained researchers. Participants were informed about the study verbally. All participants read and stated in handwriting on the informed consent form that they had read and understood the remits of the study; they then signed the informed consent form. The questionnaire consisted of five parts. The first part included Health Literacy Scale-Short Form (HLS-SF12). This scale was developed by Tuyen V. Duong et al. (18). A validity and reliability study of the Turkish version of the scale was conducted by Karahan and Eskici (19) and the Cronbach Alpha coefficient of the Turkish version was found to be 0.887.

The scale included 4-point Likert type response options ranging from 1 (very difficult) to 4 (very easy) and consisted of 12 items. The second part, which included the Digital Healthy Diet Literacy Scale (DDL scale), was developed by Tuyen V. Duong (20).

The scale included 4-point Likert-type answer options and consisted of 4 items (20). A validity and reliability study of the Turkish version of the scale was conducted by Karahan and Eskici (19) and the Cronbach Alpha coefficient of the Turkish version was found to be 0.839. In the third part, questions were asked about the participants’ internet and social media usage and, in the fourth part, general information questions were asked. Questions regarding internet and social media usage and general information forms were developed for this study. Height and body weight were taken according to the participants’ declaration. In the fifth part, the Mediterranean Diet Adherence Scale (MEDAS) was used to determine the adherence of the individuals participating in the study with the Mediterranean diet (21). As a result of the scale, it is concluded whether the individual has Mediterranean-type eating habits. The validity and reliability study of MEDAS for adaptation to Turkish society was carried out by Pehlivanoğlu et al. (22). MEDAS has been evaluated by a dietitian.

### Scoring

***HLS-SF12***: The formula (Index = (Average-1) × 50/3) is used in the evaluation of the scale. The average is calculated by dividing the total score of the scale by the number of scale items. The HL index value calculated by the formula varies between 0 and 50, with a higher score indicating better HL (18).

***DDL Scale***: The formula (Index = (Average-1) × 50/3) is used in the evaluation of the scale. The average is calculated by dividing the total score of the scale by the number of scale items. The DDL index value calculated by the formula varies between 0 and 50, with a higher score indicating better DDL (20).

***MEDAS***: In the scale consisting of 14 questions, each question is evaluated as 0 or 1 point depending on the answer given. Individuals with higher scores are considered as having higher compliance with the Mediterranean diet. A total of ≥7 points in the total scores of the answers to the questions indicate adherence with the Mediterranean diet (21).

***Self-assessed Health***: Evaluation was applied by using a scale where 1: Excellent, 2: Very good, 3: Good, 4: Bad, and 5: Very bad.

**Figure 1:**
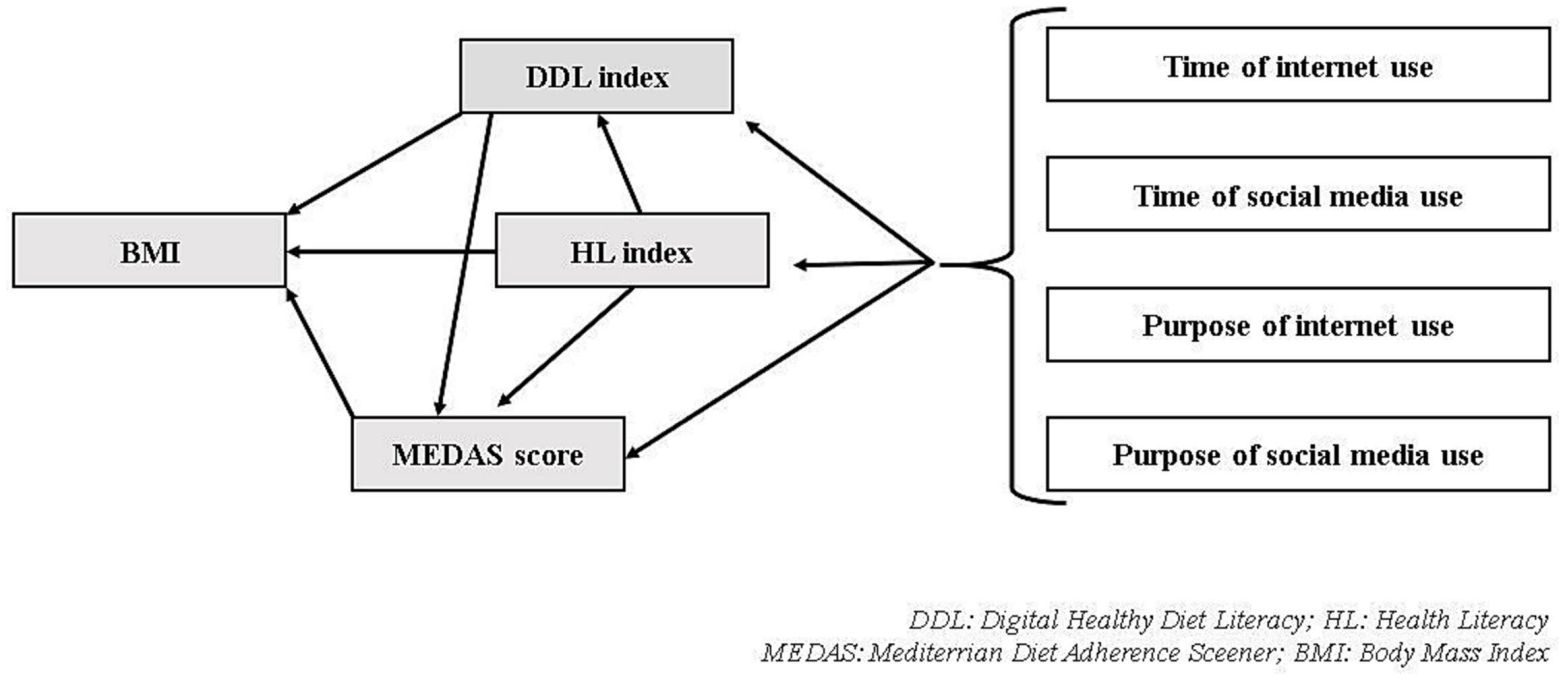
Variables examined relationally within the purpose of the study. DDL, Digital Healthy Diet Literacy; HL, Health Literacy; MEDAS, Mediterranean Diet Adherence Screener; BMI, Body Mass Index.

The study investigated the HL and DDL levels and the relationship between HL, DDL, and MEDAS scores. In addition, the effect of time of internet/social media use and purpose of internet/social media use on HL index, DDL index, and MEDAS score was analyzed (Figure 1).

### Statistical analysis

IBM’s SPSS software version 24.0 (IBM Corp., Armonk, NY, United States) was used to analyze data. Kurtosis and skewness values were examined to evaluate normality and values between 1.5 and +1.5 were considered to be normally distributed (23). Descriptive statistics were used to analyze general characteristics. To examine differences between participants’ characteristics (daily social media usage time, number of actively used social media accounts, purpose of social media use, Mediterranean diet adherence, etc.) and HL and DDL index score, a *t*-test or analysis of variance (ANOVA) was performed. Tukey’s test was used as a complement to determine the differences resulting from one-way analysis of variance. Bivariate correlation analyses (Pearson Correlation Analysis) were performed in which the statistical correlations of HL index, DDL index, MEDAS score, age, BMI (Body Mass Index), internet and social media use time (Figure 1). Linear regression models were created to identify factors associated with HL index, DDL index, MEDAS score, and BMI. The path analysis of the study was performed in the AMOS program. The maximum likelihood method was used. The model’s fitting was evaluated using the Root Mean Square Error of Approximation (RMSEA), Tucker-Lewis Index (TLI), and NFI. The findings were evaluated at a 95% confidence interval and a 5% significance level.

## Results

**Figure 2:**
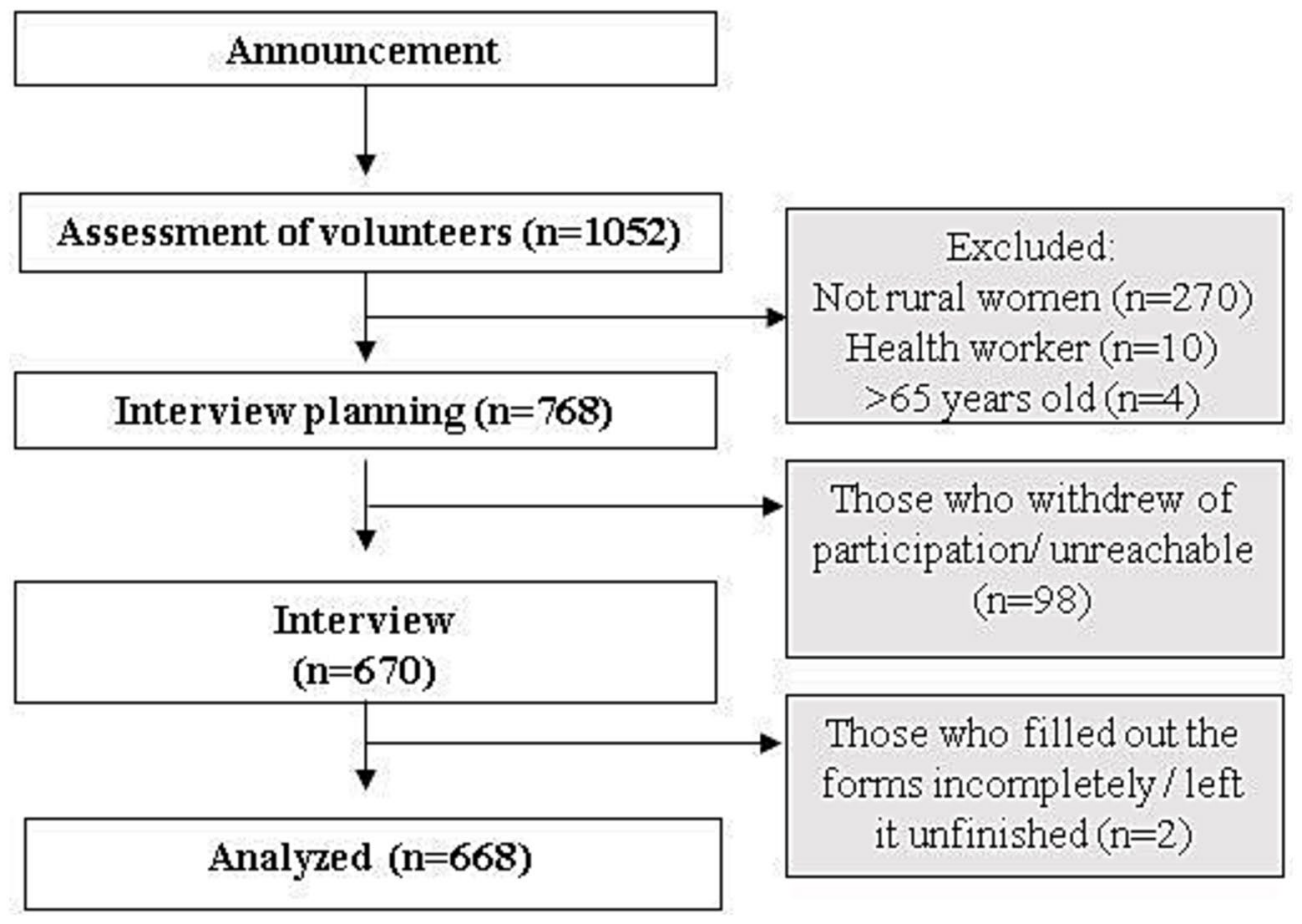
Participant selection flowchart.

In total, 1,052 volunteers were evaluated according to the inclusion–exclusion criteria until the targeted sample size of 768 was reached. Of these, 270 were not rural women, 10 were healthcare professionals, and 4 were over 65 years of age and therefore were not included in the study. 98 of 768 volunteers withdrew from the study or could not be reached on the scheduled interview day. Two volunteers withdrew before completing the questionnaire. The study was completed with 668 people. The participant selection flow chart is shown in Figure 2. Participants’ provinces are shown in Supplementary Table S1.

The mean age of the participants is 36.05 ± 11.57 years, the mean BMI is 25.02 ± 4.18 kg/m2, the mean number of actively used social apps is 2.94 ± 1.08, the mean MEDAS score is 5.90 ± 2.19, and the average personal health assessment score is 2.94 ± 0.70 (1: very bad, 5: excellent). Characteristics of the participants’ general information are given in Table 1.

**Table 1:** Characteristics of the participants.

Characteristics		n	Percent (%)
Marital status	Single	237	35.5
	Married	391	58.5
	Widow/Divorced	40	6.0
	Total	668	100.0
Job	Housewife	230	34.4
	Farmer	17	2.5
	Animal husbandry	14	2.1
	Employee	235	35.1
	Artisan	21	3.1
	Student	108	16.2
	Retired	15	2.2
	Others	28	4.2
	Total	668	100.0
Smoking	Non-smoker	358	53.6
	Smoker	84	12.6
	Quit smoking	226	33.8
	Total	668	100.0
Alcohol use	Almost every day	7	1.0
	2 times a week	27	4.0
	Once a month	105	15.7
	Never	529	79.2
	Total	668	100.0

HL and DDL index vary depending on the time of internet use, purpose of internet use, time of social media use, purpose of social media use, education, BMI, and MedDiet (Mediterranean Diet) adherence. According to the post-Hoc test, the DDL index of the participants whose daily internet usage time is <1 h and 1–3 h are statistically similar (*p* = 0.261) and they are significantly lower than all other groups. However, 1–3 h is not significantly different from >9 h (*p* = 0.055). The HL index scores of those who use the Internet mostly for gaming purposes are statistically significantly lower than all other groups and the DDL index are statistically significantly lower than those who use the Internet for Social network use, Communication, Reading/watching the news, Listening to music/radio/podcast, and Research. The DDL index of those who use social media for less than 1 h are statistically significantly lower than all other groups. HL index is statistically significantly lower in groups <1 h and 1–3 h than all other groups. In addition, the DDL index of those who use social media for 1–3 h is statistically significantly lower than those who use social media for 4–6 h or 7–9 h. The DDL index of those who use social media to follow their friends’ posts and watch short videos/reels is statistically significantly lower than those who use it to follow the news and share photos/videos/texts. The HL index of those who use social media to follow their friends’ posts is statistically significantly lower than all other groups. The DDL index and HL index of university graduates are statistically significantly higher than all other groups. In addition, the DDL and HL index scores of secondary school graduates are statistically significantly lower than those of high school and university graduates. The DDL index of overweight individuals are significantly lower than all other groups except obese individuals (*p* = 0.09). The HL index scores of overweight individuals are significantly lower than all other groups. In self-assessed health, the DDL index of those who reported their health status as ‘bad’ was found to be statistically lower than all other groups, and excellent was statistically higher than those who reported their health status as good and very good. In self-assessed health, the HL index of those who reported their health status as ‘bad’ was found to be statistically lower than all other groups and ‘good’ was higher than ‘excellent’ (*p* = 0.001). The DDL index and HL index are higher in those who comply with the Mediterranean diet (respectively *p* < 0.001, *p* < 0.001). The HL index of those with chronic disease is lower than those without (*p* = 0.033). Both HL and DDL indexes are higher in those with a previous diet history under the supervision of a dietitian (*p* < 0.001). The HL and DDL indexes of participants whose household income is higher than their expenses are the highest, while those whose household income is lower than their expenses are the lowest (See Table 2). The results of all post-Hoc tests for factors affecting the HL index and DDL index levels are presented in Supplementary Table S2.

**Table 2:** Factors affecting the HL index and DDL index of the rural female population.

Characteristics		DDL index				HL index		
		N	Mean	Sd	*p*	Mean	Sd	*p*
Time of internet use	<1 h	82	16.51	12.55	<0.001*	22.44	9.84	<0.001^a^^,^**
	1-3 h	294	19.77	12.58		23.84	10.66	
	4–6 h	176	25.09	12.77		29.19	10.25	
	7–9 h	69	27.83	12.85		33.47	7.42	
	>9 h	47	25.27	16.40		32.80	10.56	
	Total	668	21.99	13.40		26.70	10.84	
Purpose of internet use	Social media	373	21.28	12.92	<0.001*	24.93	11.05	<0.001^a^^,^**
	Communication	111	25.53	14.91		29.75	10.10	
	Shopping	54	24.85	12.97		32.20	9.35	
	Gaming	42	14.29	9.74		19.47	8.64	
	Listening to music/radio/podcast	36	25.81	12.82		29.94	9.69	
	Reading/watching news	32	18.36	14.46		29.47	9.02	
	Official affairs	5	31.67	12.01		31.66	8.86	
	Others	15	20.27	11.77		31.94	7.16	
	Total	668	21.99	13.40		26.70	10.56	
Time of social media use	<1 h	158	17.61	12.83	<0.001*	23.95	10.24	<0.001^a^^,^**
	1-3 h	340	21.53	12.65		25.52	10.85	
	4–6 h	115	26.37	12.88		30.33	9.67	
	7–9 h	40	30.00	13.71		33.82	8.54	
	>9 h	15	23.61	19.83		34.44	12.99	
	Total	668	21.99	13.40		26.70	10.56	
Purpose of social media use	Follow news	213	25.95	12.88	<0.001*	31.35	8.65	<0.001^a^^,^**
	Following friends	189	18.21	12.84		21.09	10.77	
	Watching short videos video/reels	136	19.24	12.00		25.12	10.70	
	Communication	71	22.18	15.23		26.91	11.58	
	Sharing photo/video/text	52	26.04	12.59		30.95	7.98	
	Others	7	25.00	17.17		31.74	10.34	
	Total	668	21.99	13.40		26.70	10.56	
Education	Literate	43	18.22	13.63	<0.001*	24.09	12.17	<0.001^a^^,^**
	Primary school	101	18.23	13.58		21.91	11.56	
	Secondary school	67	15.05	10.92		19.44	8.91	
	High school	214	22.12	13.31		26.99	10.43	
	University	243	26.03	12.64		30.91	9.05	
	Total	668	21.99	13.40		26.70	10.56	
BMI	Underweight	34	26.71	12.39	<0.001*	32.47	9.01	<0.001^a^^,^**
	Normal weight	305	23.87	13.44		28.88	10.42	
	Overweight	256	18.84	12.34		23.06	10.75	
	Obese	73	22.94	15.11		27.70	9.88	
	Total	668	21.99	13.40		26.70	10.56	
MedDiet adherence	No (MEDAS score <7)	407	18.94	12.81	<0.001**	23.72	10.80	<0.001**
	Yes (MEDAS score ≥7)	261	26.74	12.93		31.35	9.13	
	Total	668	21.99	13.40		26.70	10.56	
Self-assessed health	Excellent	29	30.45	15.43	<0.001*	34.77	9.00	<0.001^a^^,^**
	Very good	91	24.54	12.82		29.59	10.25	
	Good	448	21.65	13.17		26.63	10.67	
	Bad	90	17.63	12.38		21.08	9.87	
	Very bad	10	28.75	15.76		31.11	8.70	
	Total	668	21.99	13.40		26.70	10.56	
Chronic disease	Yes	189	21.03	13.96	0.244	25.29	10.92	0.033^b^^,^*
	No	479	22.37	13.17		27.27	10.76	
	Total	668	21.99	13.40		26.70	10.56	
Diet history under the supervision of a dietitian	Yes	195	24.21	13.43	0.006**	28.29	10.40	0.013^b^^,^*
	No	473	21.08	13.29		26.05	10.95	
	Total	668	21.99	13.40		26.70	10.56	
Household income	Lower than expenses	195	18.74	13.04	<0.001*	22.58	10.53	<0.001^a^^,^**
	Equal to expenses	339	22.31	13.72		27.56	10.85	
	Higher than expenses	134	25.93	11.93		30.57	9.25	
	Total	668	21.99	13.40		26.70	10.56	

a One-way Anova.

b t test.

HL İNDEX, Health literacy Index; DDL index, Digital Healthy Diet Literacy Index; *p < 0.05; **p < 0.001.

The relationships between participants’ characteristics, such as the purpose and duration of internet and social media use, Mediterranean diet compliance, and HL and DDL index, are presented in Table 3. Analysis results revealed a high positive correlation (*r* = 0.686, *p* < 0.001) between HL and DDL index. A statistically significant correlation was determined between adherence to the Mediterranean diet: moderately with the HL index and weakly positively with the DDL index. In addition, the DDL index had a weak negative correlation with age and BMI, and the HL index had a weak positive correlation with internet usage time, social network usage time, and number of social networks.

**Table 3:** Correlation of HL index, DDL index, and MEDAS with other variables.

Variables		HL index	DDL index	MEDAS score
HL index	*r*	1	0.685**	0.423**
	*p*		<0.001	<0.001
DDL index	*r*	0.685**	1	0.345**
	*p*	<0.001		<0.001
MEDAS Score	*r*	0.423**	0.345**	1
	*p*	<0.001	<0.001	
BMI	*r*	−0.199**	−0.133**	−0.047
	*p*	<0.001	0.001	0.228
Time of internet use	*r*	0.323**	0.228**	0.172**
	*p*	<0.001	<0.001	<0.001
Number of social media account	*r*	0.098*	0.070	−0.006
	*p*	0.011	0.072	0.886
Time of social media use	*r*	0.258**	0.203**	0.096*
	*p*	<0.001	<0.001	0.013
Age	*r*	−0.278**	−0.157**	−0.067
	*p*	0.000	0.000	0.084
Self-assessed health score	*r*	−0.155**	−0.224**	−0.132**
	*p*	< 0.001	< 0.001	0.001

*p < 0.05 level; **p < 0.001 level (2-tailed); HL index, Health literacy Index; DDL index, Digital Healthy Diet Literacy Index; MEDAS, Mediterranean Diet Adherence Screener.

Regression analysis models performed on HL index, DDL index, and MEDAS scores are given in Table 4.

**Table 4:** Linear regression analyses on HL index, DDL index, and MEDAS score.

Model no	Independent variable	Dependent variable	B	Std error	*β*	*t*	R	R^2^	F	*p*
1	HL index	DDL index	0.466	0.375	0.685	1.244	0.685	0.469	587.361	<0.001**
2	HL index	MEDAS Score	2.192	0.318	0.423	6.898	0.422	0.179	144.939	<0.001**
3	DDL index	MEDAS Score	4.662	0.153	0.345	30.412	0.345	0.119	89.823	<0.001**
4	HL index	BMI	28.348	0.655	−0.199	43.283	0.199	0.040	27.448	<0.001**
5	DDL index	BMI	25.931	0.309	−0.133	84.055	0.133	0.018	11.987	<0.001**

*p < 0.05 level; **p < 0.001 level; HL İNDEX, Health literacy Index; DDL index, Digital Healthy Diet Literacy Index; MEDAS, Mediterranean Diet Adherence Screener.

**Model 1**: It is the regression analysis model for predicting the health literacy index and the Digital Healthy Diet Literacy Index. Since *p* < 0.05, the model is significant. The R2 value, which is expressed as the explanatory power of the model, was calculated as 0.469 (R = 0.685; *p* < 0.001). This value shows that 46.9% of the DDL index variable is explained by the independent variable in the model, namely the HL index. The beta coefficient of the independent variable included in the regression analysis is 0.685 (*p* < 0.001). Accordingly, the HL index has a significant effect on the DDL index.

**Model 2**: It is the regression analysis model of the health literacy index predicting the Mediterranean diet compliance score. Since *p* < 0.005, the model is significant. The R2 value expressing the explanatory power of the model was calculated as 0.179 (R = 0.422; *p* < 0.001). This value shows that 17.9% of the MEDAS score variable is explained by the independent variable in the model, namely the HL index. The beta coefficient of the independent variable included in the regression analysis is 0.423 (*p* < 0.001). Accordingly, the HL index has a significant effect on the MEDAS score.

**Model 3**: This is the regression analysis of the digital healthy diet literacy index predicting the Mediterranean diet compliance score. Since *p* < 0.05, the model is significant. The R2 value expressed as the explanatory power of the model was calculated as 0.119 (R = 0.422; *p* < 0.001). This value shows that 11.9% of the MEDAS score variability is explained by the independent variable in the model, namely the DDL index. The beta coefficient of the independent variable included in the regression analysis is 0.345 (*p* < 0.001). Accordingly, the DDL index has a significant effect on the MEDAS score.

**Model 4**: It is the regression analysis model for predicting the health literacy index and BMI. Since *p* < 0.05, the model is significant. The R2 value, which is expressed as the explanatory power of the model, was calculated as 0.040 (R = 0.199; *p* < 0.001). This value shows that 4% of the BMI variable is explained by the independent variable in the model, namely the HL index. The beta coefficient of the independent variable included in the regression analysis is −0.199 (*p* < 0.001). Accordingly, the HL index has a significant effect on BMI.

**Model 5**: It is the regression analysis model for predicting the Digital Healthy Diet Literacy Index and BMI. Since *p* < 0.05, the model is significant. The R2 value, which is expressed as the explanatory power of the model, was calculated as 0.018 (R = 0.133; *p* < 0.001). This value shows that 1.8% of the BMI variable is explained by the independent variable in the model, namely the DDL index. The beta coefficient of the independent variable included in the regression analysis is 0.685 (*p* < 0.001). Accordingly, the DDL index has a significant effect on BMI.

**Figure 3:**
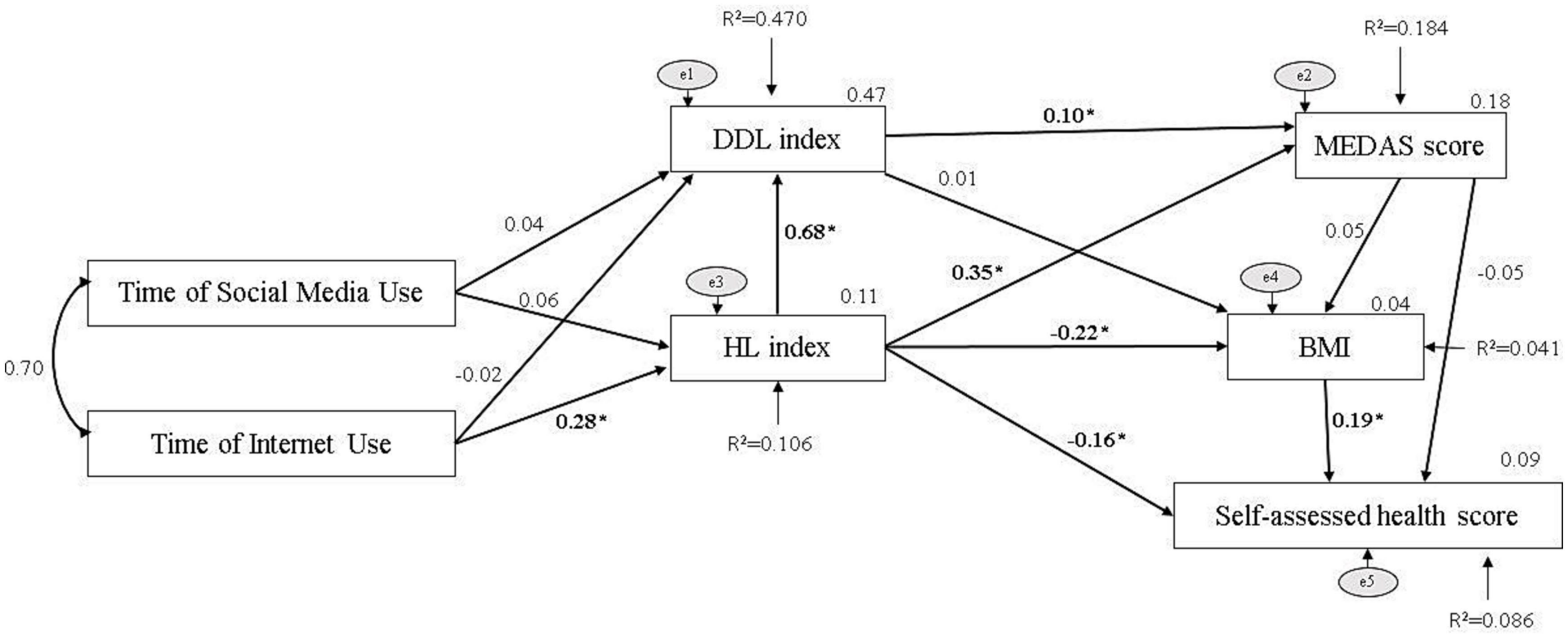
Path model of the assumptions. *indicates statistically significant. Fit indices, Root Mean Square Error of Approximation (RMSEA) = 0.035, Tucker Lewis Index (TLI) = 0.985, NFI=0.989.

In this study, path analysis was conducted to test the model created with the assumption that time on social media and internet use affect HL, DDL, BMI, Mediterranean diet adherence, and self-assessed health score. Results of goodness of fit indices of the model [Root Mean Square Error of Approximation (RMSEA) = 0.035, Tucker-Lewis Index (TLI) = 0.985, NFI = 0.989] indicated that the model was appropriate (24). According to the results of the analysis, internet use time affects HL index, and HL index affects BMI, MEDAS score, and self-assessed health score. The effect paths of time of social media use were not found to be significant. Endogenous-exogenous variables of the path analysis and detailed data are given in Figure 3.

## Discussion

The study examines the HL and DDL of rural women living in Turkey who use social media and its effects on healthy nutrition and provides valuable data in this field. It was found that HL and DDL index differ depending on the duration of internet use, purpose of internet and social media use, household income, and education level. In addition, it was determined that DDL and HL index correlated with MEDAS score and BMI.

The healthcare system includes not only the treatment of diseases but also the protection and promotion of health; in this context, it is possible to say that all individuals should have health knowledge. The healthcare system is constantly developing and changing, therefore it is a necessity to follow the developments. To protect and maintain health, it is necessary to have the ability to access health information, understand it correctly, and use this information (25). Health literacy was first used by Scott Simonds in 1974 and has been developed over the years. The definition has been updated in the Healthy People 2030 initiative published by the US government (26). The current definition considers HL under two headings: ‘personal health literacy’ and ‘institutional health literacy’. In the study, personal health literacy, defined as “the degree to which individuals have the ability to find, understand, and use information and services to inform health-related decisions and actions for themselves and others” in the relevant report, was examined. The first large-scale research in Türkiye was conducted in 2014 using the Health Literacy Survey-European Union (HLS-EU). Researchers showed in the study that only one-third of Türkiye has adequate/excellent HL levels (27).

In a study examining only women, it was shown that the HL of 45.9% of the participants was determined as insufficient, 30.6% as limited, 16% as sufficient, and 7.4% as excellent by using TSOY-32 (28). Studies conducted in developing countries such as South Africa, Malaysia, and Taiwan have shown that rural individuals have lower HL (29–32). Guçlu et al. (33) reported that HL in the rural population, examined by use of HLS-EU-Q47, was insufficient in 70.9% of the participants and problematic in 20.6% of the participants. In this study, the HL index score that can be obtained from this index is between 0 and 50, and a higher score indicates a better HL. The average HL index was 26.70. This value can be interpreted as the participants’ HL being at a moderate level.

HL can be affected by many factors such as age group, education level, income level, gender, presence of chronic disease, and cognitive status (34–39). In a systematic review study examining Iranian women, the HL score of women with chronic diseases was found to be significantly lower. Additionally, a significant relationship has been shown between HL and self-efficacy and self-care behaviors (39). In this study, it was determined that HL was lower in people with chronic diseases (*p* = 0.033).

Similar to the literature, in this study, age, education level, and income level affect HL (22, 29–33). In the results, it was shown that age and HL and DDL are negatively correlated (respectively; *r* = −0.277, *p* < 0.001; *r* = −0.158, *p* < 0.001), that those with a high school education or above have higher HL and DDL, and that those with a higher household income have higher HL and DDL.

There are studies showing that HL may affect health status and healthy behaviors (40–48). During the recent global COVID-19 pandemic, it was observed that social media was used as a source of health information and had a crucial role in disseminating health information and combating information epidemics and misinformation (49–58). For this reason, studies examining factors such as age, gender, region of residence, ethnicity, lifestyle, sources of information, mass media, social media channels, and diseases that may affect HL would benefit public health. In addition, it has been confirmed that parental HL, as well as socioeconomic level, affects health and well-being in children, such as healthy nutrition and exercise (59). Therefore, it can be considered that improving HL can serve to create a healthy society.

HL may be lower in individuals who do not use the Internet (28). It has been shown that there is a strong relationship between HL and internet access and use, and internet use has the potential to improve HL (12, 60–62). In this study, since our research sample consists of participants who use the Internet and social media, HL index was determined to be lower than we expected. It is possible to explain this situation in the findings we obtained as a result of our studies. Participants reported that they mostly use the Internet to use social media (55.84%) and social media to follow current affairs (31.89%). None of the participants reported that the main purpose of using the internet or social media was to obtain health information. It shows that internet access and use, as well as the purpose and duration of internet and social media use, have the potential to affect HL and DDL.

In studies focusing on the effects of HL on nutritional intake, there are studies that link HL with salt intake/awareness, sugar and fat consumption, vegetable and fruit consumption, and physical activity, as well as studies that do not establish a significant relationship (40, 43, 46, 63, 65).

Improvement of HL and DL may also increase the tendency to improve nutritional behavior (60). In this study, both HL and DDL were determined to be correlated with MEDAS. In a systematic review study that included 39 studies, HL skills and knowledge were shown to be effective in obesity and BMI management (66). A meta-analysis examining 33 studies reported that HL may be effective in helping individuals to lose weight and improve physical activity (67). Similar results were obtained in this study: DDL and HL index scores of overweight individuals were found to be significantly lower than all other groups (*p* < 0.05), and BMI was found to be negatively correlated with both HL and DDL index.

None of the existing mass media offers the consumer similar interactive opportunities as social media. It is known that the majority of smartphone owners use their phones, especially social media, to obtain information about health conditions. This shows that social media has the potential to influence health outcomes through HL, health promotion, and health communication interventions (3, 9, 68). Although it is known that social media has become an important way of learning, the mechanisms that explain the effects of social media use on knowledge are not yet clear. Jiang et al. explained the pathways through which social media use is linked to health information by the cognitive mediation model. This model is implemented by considering different patterns of information acquisition (media attention, information discussion, etc.), information processing (elaboration, etc.), and information seeking experience. Using this model, a cross-sectional study conducted in China has shown that paying attention to health information on social media has a direct and positive relationship with health knowledge (69).

It has been reported that the increase in HL and nutrition literacy in the digital environment can also increase the quality of life of individuals (70). In this study, the HL index of those who reported their health status as “bad” was found to be statistically lower than all other groups, and the DDL index was statistically lower than those who reported their health status as very good and excellent (*p* < 0.05). The self-assessed health status that was used in this study is based on the principle of an individual’s qualitative assessment of their own health status. This subjective nature may cause the tool to be questioned for bias. However, perceived health status assessment is a determining factor for a person’s need for healthcare, so it is accepted that self-assessed health status data can provide data for public health planning (71). In addition, it has significant public health benefits due to its ease of use, affordability, and predictability of health status (72).

The study has some limitations. The sample number was distributed proportionally to the rural female population of the selected provinces. However, it does not allow comparison between regions. Sample selection is limited to people to whom study announcements reach. The study focused on the relationship between HL, DDL, and healthy eating behaviors and their relationship with the purpose and duration of internet/social media use. However, many factors may affect HL, DDL, and feeding behavior. Studies on the relationship between HL and DDL levels and the nutritional behavior of rural women using social media in Türkiye are limited. Therefore, our results need to be confirmed with further studies. The study also has some strengths. Conducting face-to-face interviews, applying and evaluating MEDAS by a dietician, and having the interviews conducted by trained researchers increase the reliability of the data. In addition, inviting twice the number of volunteers to the minimum number needed ensured the representativeness of the sample and reduced the risks that may arise from data loss.

## Conclusion

In light of the results, it is possible to say that the HL and DDL of rural women using social media in Türkiye are at a moderate level. The HL index and DDL index show a positive correlation with the MEDAS score. In addition, the HL and DDL indexes differ depending on the duration and purpose of internet use, age, presence of chronic disease, education level, and income level. It is recommended to conduct further studies evaluating the use of social media as a tool to promote HL, DDL, and healthy eating behaviors in different populations and subsequent studies examine the mechanisms of influence of social media use on HL and DDL.

## Data Availability

The raw data supporting the conclusions of this article will be made available by the authors, without undue reservation.
