# Supplementary material for: Understanding health literacy and digital healthy diet literacy in rural women in Türkiye: a cross-sectional study on social media use and Mediterranean diet adherence
Source: Front Public Health. 2025 May 30;13:1559159. doi: 10.3389/fpubh.2025.1559159 (PMC12164640; doi:10.3389/fpubh.2025.1559159)
Supplement: Supplementary file 1 [file Table_1.docx]

**Table S1.** Province of the participants

|  | | n | Percent (%) |
| --- | --- | --- | --- |
| Province | Ağrı | 81 | 12.1 |
|  | Afyon | 125 | 18.7 |
|  | Batman | 46 | 6.9 |
|  | Bitlis | 53 | 7.9 |
|  | Düzce | 54 | 8.1 |
|  | Edirne | 42 | 6.3 |
|  | Giresun | 60 | 9.0 |
|  | Karaman | 25 | 3.7 |
|  | Osmaniye | 47 | 7.0 |
|  | Tokat | 77 | 11.5 |
|  | Yozgat | 58 | 8.7 |
|  | Total | 668 | 100.0 |
