# Supplementary material for: Understanding health literacy and digital healthy diet literacy in rural women in Türkiye: a cross-sectional study on social media use and Mediterranean diet adherence
Source: Front Public Health. 2025 May 30;13:1559159. doi: 10.3389/fpubh.2025.1559159 (PMC12164640; doi:10.3389/fpubh.2025.1559159)
Supplement: Supplementary file 2 [file Table_2.docx]

**Table S2.** Factors affecting the HLindex and DDL index levels of the rural female population

|  | | **DDL index** | | | | **HL index** | | |
| --- | --- | --- | --- | --- | --- | --- | --- | --- |
|  | | N | Mean | sd | p | Mean | Sd | p |
| Time of internet use | <1 hour | 82 | 16.51 | 12.55 | <0.001* | 22.44 | 9.84 | <0.001^a^** |
|  | 1-3 hours | 294 | 19.77 | 12.58 |  | 23.84 | 10.66 |  |
|  | 4-6 hours | 176 | 25.09 | 12.77 |  | 29.19 | 10.25 |  |
|  | 7-9 hours | 69 | 27.83 | 12.85 |  | 33.47 | 7.42 |  |
|  | >9 hours | 47 | 25.27 | 16.40 |  | 32.80 | 10.56 |  |
|  | Total | 668 | 21.99 | 13.40 |  | 26.70 | 10.84 |  |
| ***DDL index Post Hoc p values****: <1 hours/ 4-6 hours p< 0.001; <1 hours/7-9hours p<0.001; <1 hours/> 9 hours p= 0.002; 1-3 hours/4-6 hours p< 0.001; 1-3 hours/7-9 hours p<0.001; other p values >0.05.* ***HL index Post Hoc p Values****: <1 hours/ 4-6 hours p< 0.001; <1 hours/7-9hours p<0.001; <1 hours/> 9 hours p<0.001; 1-3 hours/4-6 hours p< 0.001; 1-3 hours/7-9 hours p<0.001; 1-3 hours/>9 hours p<0.001; /4-6 hours//6-9 hours p=0.026; other p values >0.05.* | | | | | | | | |
| Purpose of internet use | Social media | 373 | 21.28 | 12.92 | <0.001* | 24.93 | 11.05 | <0.001^a^** |
|  | Communication | 111 | 25.53 | 14.91 |  | 29.75 | 10.10 |  |
|  | Shopping | 54 | 24.85 | 12.97 |  | 32.20 | 9.35 |  |
|  | Gaming | 42 | 14.29 | 9.74 |  | 19.47 | 8.64 |  |
|  | Listening music/radio/podcast | 36 | 25.81 | 12.82 |  | 29.94 | 9.69 |  |
|  | Reading/watching news | 32 | 18.36 | 14.46 |  | 29.47 | 9.02 |  |
|  | Official affairs | 5 | 31.67 | 12.01 |  | 31.66 | 8.86 |  |
|  | Others | 15 | 20.27 | 11.77 |  | 31.94 | 7.16 |  |
|  | Total | 668 | 21.99 | 13.40 |  | 26.70 | 10.56 |  |
| ***DDL index Post Hoc p values:*** *Social Media/Communication: p=0.003; Social Media/Listening Music/Radio/Podcast p=0.049; Social Media/Gaming p=0.001; Communication/Reading-Watching News p=0.007; Communication/Gaming p<0.001), Reading-Watching News/Shopping p=0.027; Reading-Watching News/Official Affairs p=0,035; Reading-Watching News/Listening Music-Radio-Podcast p=0.020; Listening Music/Radio/Podcast/Gaming p<0.001; Gaming/ Official Affairs p=0.005; Gaming / Shopping p<0.001; other p values >0.05.* ***HL index Post Hoc p Values****: Social Media/ Communication p=0.001; Social Media/Gaming p=0.026; Social Media/Shopping p<0.001; Communication/Gaming p<0.001; Reading-Watching News/Gaming p=0.001; Listening music/radio/podcast/Gaming p<0.001; other p values >0.05.* | | | | | | | | |
| Time of social media use | <1 hour | 158 | 17.61 | 12.83 | <0.001* | 23.95 | 10.24 | <0.001^a^** |
|  | 1-3 hours | 340 | 21.53 | 12.65 |  | 25.52 | 10.85 |  |
|  | 4-6 hours | 115 | 26.37 | 12.88 |  | 30.33 | 9.67 |  |
|  | 7-9 hours | 40 | 30.00 | 13.71 |  | 33.82 | 8.54 |  |
|  | >9 hours | 15 | 23.61 | 19.83 |  | 34.44 | 12.99 |  |
|  | Total | 668 | 21.99 | 13.40 |  | 26.70 | 10.56 |  |
| ***DDL index Post Hoc p values:*** *<1 hour/1-3 hours p=0.02; <1 hour/4-6 hours p<0.001; <1 hour/7-9 hours p<0.001; 1-3 hours/<4-6 hours p=0.001; 1-3 hours/7-9 hours p<0.001; other p values >0.05.* ***HL index Post Hoc p Values:*** *<1 hour/4-6 hours p<0.001; <1 hour/7-9 hours p<0.001; <1 hour/ >9 hours p=0.002; 1-3 hours/<4-6 hours p<0.001; 1-3 hours/7-9 hours p<0.001;1-3 hours/<9 hours p=0.012; other p values >0.05.* | | | | | | | | |
| Purpose of social media use | Follow agenda | 213 | 25.95 | 12.88 | <0.001* | 31.35 | 8.65 | <0.001^a^** |
|  | Following friends | 189 | 18.21 | 12.84 |  | 21.09 | 10.77 |  |
|  | Watching short videos video/reels | 136 | 19.24 | 12.00 |  | 25.12 | 10.70 |  |
|  | Communication | 71 | 22.18 | 15.23 |  | 26.91 | 11.58 |  |
|  | Sharing photo/video/text | 52 | 26.04 | 12.59 |  | 30.95 | 7.98 |  |
|  | Others | 7 | 25.00 | 17.17 |  | 31.74 | 10.34 |  |
|  | Total | 668 | 21.99 | 13.40 |  | 26.70 | 10.56 |  |
| ***Post Hoc p values:*** *Sharing photo/video/text/Following friends p=0.002; Sharing photo-video-text/Watching short videos video-reels p=0.017; Following friends/ Follow agenda p<0.001; Follow agenda/ Watching short videos video/reels p<0.001;* *other p values >0.05.* ***HL index Post Hoc p Values:*** *Sharing photo/video/text/Following friends p<0.001; Follow agenda/ Following friends p<0.001; Following friends/Comminication p<0.001; Following friends/* *Watching short videos video/reels p=0.005; Follow agenda/Comminication p=0.028; / Follow agenda/* *Watching short videos video/reels p<0.001;* *Sharing photo/video/text/ Following friends p<0.001; Sharing photo/video/text/* *Watching short videos video/reels p=0.005; other p values >0.05.* | | | | | | | | |
| Education | Literate | 43 | 18.22 | 13.63 | <0.001* | 24.09 | 12.17 | <0.001^a^** |
|  | Primary school | 101 | 18.23 | 13.58 |  | 21.91 | 11.56 |  |
|  | Secondary school | 67 | 15.05 | 10.92 |  | 19.44 | 8.91 |  |
|  | High school | 214 | 22.12 | 13.31 |  | 26.99 | 10.43 |  |
|  | University | 243 | 26.03 | 12.64 |  | 30.91 | 9.05 |  |
|  | Total | 668 | 21.99 | 13.40 |  | 26.70 | 10.56 |  |
| ***DDL index Post Hoc p values:*** *Literate/University p=0.003; Primary school/University 0.001; Secondary school/University p=0.001; High school/Secondary school p=0.001; p other p values >0.05.* ***HL index Post Hoc p Values:*** *Literate/University p<0.001; Primary school/ High school p<0.001; Primary school/ University p<0.001; Secondary school/ High school p<0.001; High school/University p<0.001; University/ Secondary school p<0.001; other p values >0.05.* | | | | | | | | |
| BMI | Underweight | 34 | 26.71 | 12.39 | <0.001* | 32.47 | 9.01 | <0.001^a^** |
|  | Normal weight | 305 | 23.87 | 13.44 |  | 28.88 | 10.42 |  |
|  | Over weight | 256 | 18.84 | 12.34 |  | 23.06 | 10.75 |  |
|  | Obese | 73 | 22.94 | 15.11 |  | 27.70 | 9.88 |  |
|  | Total | 668 | 21.99 | 13.40 |  | 26.70 | 10.56 |  |
| ***DDL index Post Hoc p values:*** *Overweight/underweight p=0.006; Overweight /Normal p<0.001; other p values >0.05.* ***HL index*** ***Post Hoc p Values:*** *Overweight/underweight p<0.001; Overweight /Normal p<0.001; Obese/ Overweight p=0.005; other p values >0.05* | | | | | | | | |
| MedDiet adherence | No(<7 points) | 407 | 18.94 | 12.81 | <0.001** | 23.72 | 10.80 | <0.001** |
|  | Yes(≥7 points) | 261 | 26.74 | 12.93 |  | 31.35 | 9.13 |  |
|  | Total | 668 | 21.99 | 13.40 |  | 26.70 | 10.56 |  |
| Self-assessed health | Excellent | 29 | 30.45 | 15.43 | <0.001* | 34.77 | 9.00 | <0.001^a^** |
|  | Very good | 91 | 24.54 | 12.82 |  | 29.59 | 10.25 |  |
|  | Good | 448 | 21.65 | 13.17 |  | 26.63 | 10.67 |  |
|  | Bad | 90 | 17.63 | 12.38 |  | 21.08 | 9.87 |  |
|  | Very bad | 10 | 28.75 | 15.76 |  | 31.11 | 8.70 |  |
|  | Total | 668 | 21.99 | 13.40 |  | 26.70 | 10.56 |  |
| ***DDL index Post Hoc p values****: Bad/Excellent p<0.001; Bad/Very good p<0.001; Bad/Good p=0.009; Bad/very bad p=0.012; Excellent/Good p=0.001; Excellent/Very good p=0.035; other p values >0.05.* ***HL index Post Hoc p Values:*** *Bad/Excellent p<0.001; Bad/Very good p<0.001; Bad/Good p<0.001; Bad/very bad p=0.034; Excellent/Good p=0.001; other p values >0.05.* | | | | | | | | |
| Chronic disease | Yes | 189 | 21.03 | 13.96 | 0.244 | 25.29 | 10.92 | 0.033^b^* |
|  | No | 479 | 22.37 | 13.17 |  | 27.27 | 10.76 |  |
|  | Total | 668 | 21.99 | 13.40 |  | 26.70 | 10.56 |  |
| Diet history under the supervision of a dietitian | Yes | 195 | 24.21 | 13.43 | 0.006** | 28.29 | 10.40 | 0.013^b^* |
|  | No | 473 | 21.08 | 13.29 |  | 26.05 | 10.95 |  |
|  | Total | 668 | 21.99 | 13.40 |  | 26.70 | 10.56 |  |
| Hausehold income | Lower than expenses | 195 | 18.74 | 13.04 | <0.001* | 22.58 | 10.53 | <0.001^a^** |
|  | Equal to expenses | 339 | 22.31 | 13.72 |  | 27.56 | 10.85 |  |
|  | Higher than expenses | 134 | 25.93 | 11.93 |  | 30.57 | 9.25 |  |
|  | Total | 668 | 21.99 | 13.40 |  | 26.70 | 10.56 |  |
| ***DDL index Post Hoc p values:*** *Lower than expenses /Equal to expenses p=0.008; Lower than expenses/ Higher than expenses p<0.001; Higher than expenses/ Equal to expenses p=0.02;* ***HL index Post Hoc p Values:*** *Lower than expenses /Equal to expenses p<0.00; Lower than expenses/ Higher than expenses p<0.001; Higher than expenses/ Equal to expenses p=0.014; other p values >0.05.* | | | | | | | | |

*^a^One Way Anova, bt testi; HLI: Health literacy Index; DDLI: Digital Healthy Diet Literacy Index; *p<0.05; **p<0.001*
